# Long Non-Coding RNA H19 Promotes Porcine Satellite Cell Differentiation by Interacting with TDP43

**DOI:** 10.3390/genes11030259

**Published:** 2020-02-28

**Authors:** Jingxuan Li, Wenjuan Zhao, Qianqian Li, Ziying Huang, Gaoli Shi, Changchun Li

**Affiliations:** 1 Key Laboratory of Agricultural Animal Genetics, Breeding and Reproduction of the Ministry of Education and Key Laboratory of Swine Genetics and Breeding of the Ministry of Agriculture, Huazhong Agricultural University, Wuhan 430070, Hubei, China; 15927409635@163.com (J.L.); zwj163oyouxiang@163.com (W.Z.); lqq101521@163.com (Q.L.); huangziyingtg@163.com (Z.H.); Shigaoli@webmail.hzau.edu.cn (G.S.); 2 The Cooperative Innovation Center for Sustainable Pig Production of Hubei Province, Wuhan 430070, Hubei, China

**Keywords:** H19, TDP43, MYOD, PSC differentiation

## Abstract

Long non-coding RNAs (lncRNAs) have been implicated in fundamental and diverse biological processes, including myogenesis. However, the molecular mechanisms involved in this process remain largely unexplored. This study found that H19 affected the differentiation of porcine satellite cells (PSCs) by directly binding to the DNA/RNA-binding protein TDP43. Functional analyses showed that TDP43 knockdown decreased PSC differentiation, whereas TDP43 overexpression exerted opposite effects in vitro. Furthermore, rescue experiments demonstrated that TDP43 can rescue the decrease in PSC differentiation caused by H19 knockdown. Mechanistically, H19 may act as a scaffold to recruit TDP43 to the promoters of MYOD and thereby activate the transcription of MYOD, leading to PSC differentiation. In summary, we elucidate the molecular mechanism by which H19 and TDP43 regulate myogenesis.

## 1. Introduction

Myogenesis is the process of skeletal muscle development and formation, which is a highly coordinated developmental process involving the coordinate expression of a number of myogenic regulatory factors, such as paired box 3, paired box 7, myogenic factor 5, myogenin (myog), and myogenic differentiation (MYOD) [1,2,3]. Satellite cells (SCs) reside between basement membranes and muscle sarcolemma in myofibers and play a vital role in the regeneration and differentiation of skeletal muscle [4,5]. SCs are activated by specific signals such as muscle injury, and can self-renew or enter the myogenic pathway and subsequently differentiate to restore muscles [6,7,8].

Long noncoding RNAs (lncRNAs) participate in numerous important biological processes including myogenesis. For example, linc-RAM promotes the assembly of the MyoD–Baf60c–Brg1 complex on the regulatory elements of myogenic genes by directly binding MyoD [9]. In addition, lncIRS1 regulates IRS1 expression and promotes skeletal muscle myogenesis through acting as a sponge for the miR-15 family [10]. LncRNAs usually perform different functions depending on their subcellular localization. For instance, nuclear lncRNAs play important roles in the regulation of gene transcription [11,12]. LncRNAs of this class usually function by interacting with chromatin modification complexes, transcription factors, and RNA-binding proteins [13,14,15,16,17]. Cytoplasmic lncRNAs usually exert their function through controlling mRNA stability and translation, controlling protein stability, modulating miRNA function, acting as competing endogenous RNA, and so on [18]. Many lncRNAs participate in the regulation of myogenesis by a variety of mechanisms [19,20]. For example, Sirt1 AS lncRNA, an lncRNA transcribed from the Sirt1 antisense strand, attenuates the inhibition of miR-34a to Sirt1 translation to accelerate myoblast proliferation and represses myoblast differentiation. In addition, it interacts with Sirt mRNA forming RNA duplex to promote Sirt1 translation [21].

H19 is a lncRNA that plays an essential role during myogenic differentiation [22,23,24]. It is a highly abundant transcript in skeletal muscle and it is upregulated in the process of myoblast differentiation and muscle regeneration. H19 exon1 can also encode the conserved microRNAs miR-675-3p and miR-675-5p, which promote mice skeletal muscle differentiation and regeneration by down-regulating Smad1, Smad5, and Cdc6 [25]. Our previous studies showed that H19 knockdown can decrease the expression of MYOG, MYOD, and myosin heavy chain (MYHC) after induced differentiation of porcine satellite cells (PSCs) at 24 and 36 h (Figure S1). Meanwhile, H19 can regulate the differentiation of PSCs through competitive binding with miRNA and directly binding with a cytoplasmic protein (unpublished). Considering that both are expressed in the nucleus and the cytoplasm, we inferred that H19 also regulates PSC differentiation by interacting with factors in the nucleus. In the present study, we found that H19 regulates PSC differentiation by interacting with Transactive response DNA-binding protein 43 (TDP43). In addition, H19 may act as a scaffold to recruit TDP43 to the promoter region of MYOD in PSCs and thereby activate the transcription of MYOD, leading to PSC differentiation.

## 2. Materials and Methods

### 2.1. Ethics Statement

In this study, we carried out animals care and all the experimentation according to the pre-approved guidelines from Regulation Proclamation No.5 of the Standing Committee of Hubei People’s Congress. All experimental protocols were approved by the Ethics Committee of Huazhong Agricultural University, Wuhan City, Hubei Province, P. R. China.

### 2.2. Satellite Cell Culture and Differentiation

Fresh PSCs were isolated from the skeletal muscles of 3-day-old Yorkshire male pigs. In order to improve the purity of PSCs and separate PSCs from fibroblast cells, we incubated the PSCs in an uncoated plate at 37 °C for 150 min and then transferred them to a new Matrigel-coated plate.

The isolated fresh PSCs were cultured on Matrigel (BD, Cat#356234) coated 10 cm plates (Corning Cat#430167) in PM+ medium. PM+ medium was composed of 76.5% RPMI 1640 (Life, Cat#A10491), 20% FBS, 0.5% chicken embryo extract (GEMINI, Cat#100-163P), 1% GlutaMax (Gibco, Life Technologies, USA, Cat#35050-061), 1% non-essential amino acids (Gibco, Life Technologies, USA, Cat#11140-050), 1% antibiotic-antimycotic (Gibco, Life Technologies, USA, Cat#15240-062), and 2.5 ng/mL human recombinant basic fibroblast growth factor (Gibco, Invitrogen, USA, Cat#13256029). Then, PSCs were transferred to DMEM containing 5% horse serum (HyClone, Cat#SH30074.02) to induce differentiation.

### 2.3. Plasmid Construction, Small Interfering RNA (siRNA) Synthesis, and Transfection

For the construction of H19 and TDP43 overexpression vectors, T4 DNA ligase (Takara, Japan, Cat# 6022Q) was used to clone the coding sequence of H19 and TDP43, then the sequences were inserted into pcDNA3.1(+) vector. The truncated H19 were obtained by PCR using the H19-pcDNA3.1- plasmid as a template and then were cloned into pcDNA3.1. For the MYOD promoter vector, the promoter region of MYOD was cloned into the PGL3 vector. The primers above were shown at Table S1. The RNA oligo against porcine H19 and TDP43 were purchased from GenePharma. The siRNA sequences used were as follows:

H19 siRNA:

(CCUCCUAGCUCUGACUCAATTdT; UUGAGUCAGAGCUAGGAGGTTdT)

TDP43 siRNA-1:

(CCGCCUGGUAGAAGGAAUUTTdT; AAUUCCUUCUACCAGGCGGTTdT)

TDP43 siRNA-2:

(CCCAUGGAAGACGACUGAATTdT; UUCAGUCGUCUUCCAUGGGTTdT)

TDP43 siRNA-3:

(GGAAUAUGAAACCCAGGUUTTdT; AACCUGGGUUUCAUAUUCCTTdT)

Negative control (NC):

(UUCUCCGAACGUGUCACGUTTdT; ACGUGACACGUUCGGAGAATTdT)

For cell transfection, the relevant plasmids or siRNAs were conducted with Lipofectamine 2000 (Invitrogen, New York, NY, USA) as advised by the manufacturer’s protocol.

### 2.4. RNA Extraction and qPCR Assay

We used Trizol reagent (Takara, Dalian, China) to extract total RNA from PSCs according to the manufacturer’s instructions. Then, we used the SuperScript III (Thermo Fisher, Boston, USA) first strand synthesis system to carry out cDNA synthesis for mRNA. Analysis of mRNA expression was carried out with SYBR Green Mix (CWBIO, Beijing, China, CW0957) and performed in a Roche LightCyler 480 system (Roche, Mannheinm, Germany) following the manufacturer’s instructions. The primers associated with qPCR are shown in Tables S2 and S3. As previously described, the 2-∆∆CT method was used to analyze the qPCR data [26].

### 2.5. Western Blotting Analysis

We washed PSCs in cell plates, lysed them in RIPA lysis buffer containing 1% PMSF, and collected them. We carried out immunoblotting assays according to standard procedures as previous described [1]. The primary antibodies used were anti-TDP43 (1:1000, Abclonal, Wuhan, China, Cat#A1183), anti-MYOG (1:500, Abcam, Cambridge,, UK, Cat#ab1835), anti-MYOD (1:1000, Abclonal, Wuhan, China, Cat#A0671), anti-MYHC (1:3000, Milipore, Darmstadt, Germany, Cat#05-716), and anti-β-Tubulin (Servicebio, Wuhan, China, Cat#GB13017-2). The HRP conjugated secondary antibodies (1:4000) HRP-labeled goat anti-mouse IgG (Servicebio, Wuhan, China, Cat#GB23301) and HRP-labeled goat anti-rabbit IgG (Servicebio, Wuhan, China, Cat#GB23303) were used as secondary antibodies.

### 2.6. Immunofluorescence Assay

After fixation with ice-cold 4% paraformaldehyde for 15 min, the cells were washed three times with phosphate buffer saline (PBS) and subsequently incubated in ice-cold 0.3% Triton X-100 at room temperature for 10 min. Then, the PSCs were blocked with blocking solution (3% bovine serum albumin, 0.3% Triton X-100, 10% FBS complemented with PBS) and further washed three times. Next, the PSCs were incubated in MYHC antibody (1:1000; Milipore, Darmstadt, Germany, Cat#05-716) at 4 °C for 12 h. Following three washes in PBS, the PSCs were incubated with Alexa 594-labeled donkey anti-mouse IgG antibodies (Antgene, Wuhan, China, Cat#ANT029) for 60 min. Lastly, the cells were stained with 4,6-diamino-2-phenyl indole (DAPI) for 10 min and washed with PBS 3 times. The images were captured by Leica SP8 confocal microscope.

### 2.7. RNA Pull-down Assay

After linearization of the plasmids, T7 RNA polymerase (Roche, Basel, Switzerland, Cat#10881767001) and biotin RNA labeling mix (Roche, Cat#11685597910) were used to synthesize transcripts of the H19 full-length and mutant fragments. Then the transcripts were treated with DNase I and EDTA. Meanwhile, proteins were extracted and lysed from PSCs. In vitro biotinylated RNAs (3 µg) were incubated with proteins overnight, and then pulled down the complex by streptavidin beads. The beads were washed five times with wash buffer. Then the proteins complexes associated with beads were analyzed by mass spectrometry and western blot.

### 2.8. RNA Fluorescence in Situ Hybridization (FISH)

RNA-FISH analyses in PSCs was performed using an lncRNA FISH Kit (Guangzhou RiboBio Co. Ltd., Guangzhou, China). Briefly, PSCs were fixed in 4% formaldehyde followed by permeabilization in PBS containing 0.5% Triton X-100. Then the experiments were performed using a Fluorescence in Situ Hybridization (FISH) kit (Guangzhou RiboBio Co. Ltd., Guangzhou, China). Finally a confocal laser-scanning microscope was used to examine the results.

### 2.9. Chromatin Immunoprecipitation (CHIP)

CHIP assay was performed using a CHIP Kit (Beyotime, Shanghai, China, P2078). In order to ensure the accuracy of the experiment, we used 10^7^ cells and 10 μg of antibodies against TDP43 (Abclonal, China, Cat#A1183) in each CHIP reaction. IgG was used as the negative control. Gene enrichment level was quantified using quantitative polymerase chain reaction (qPCR).

### 2.10. RNA Immunoprecipitation (RIP) Assay

EZ-Magna RIP Kit (Millipore, Boston, USA) was used to carry out RIP assays according to the manufacturer’s instructions. Briefly, RIP lysis buffer was used to lyse 10^8^ PSCs, and the lysates were incubated with 10 μg TDP43 antibody at 4℃ overnight. Then we added the protein A/G beads to pull down the RNA-protein complex. Reverse-transcription polymerase chain reactions were carried out to detect RNAs.

### 2.11. Statistical Analysis

All results were presented as mean ± standard deviation (SD). Multigroup comparisons of the means were carried out by one-way analysis of variance tests with post hoc contrasts by Student–Newman–Keuls tests. The two-tailed Student’s t-test was performed for significance analysis by using SAS software. *P* < 0.05 indicated significant difference.

Availability of data and materials: The datasets used and/or analyzed during this study are available from the corresponding author upon reasonable request.

## 3. Results

### 3.1. H19 Physically Interacts with TDP43

**Figure 1:**
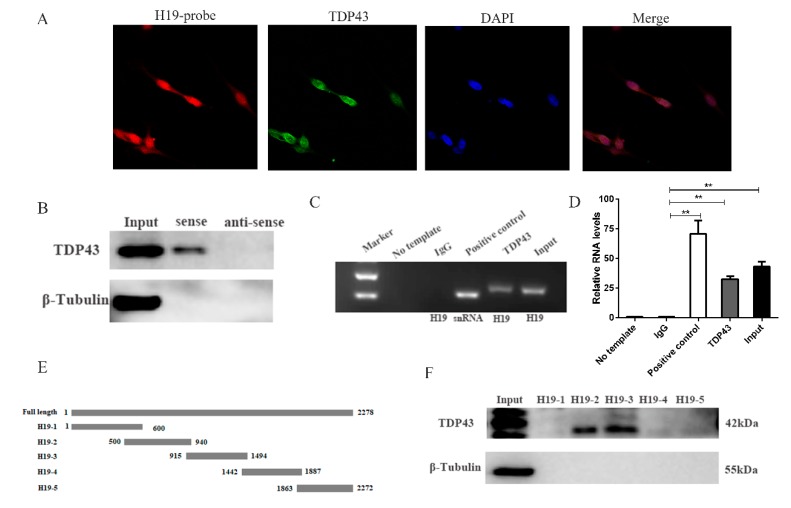
H19 physically interacts with TDP43. (**A**) Confocal Fluorescence in Situ Hybridization (FISH) images of co-localization of H19 and TDP43. (**B**) Biotin-labeled full-length H19 was used to pull down TDP43 protein. Western blotting analysis was performed to detect the TDP43 protein. β-Tubulin was used as negative control. (**C**) RNA Immunoprecipitation (RIP) assays were performed to validate the interaction between H19 and TDP43. SNRNP70 was used as positive control. The H19 and snRNA transcripts were assessed by PCR. (**D**) qPCR result of RIP assay. (**E**) The location of the H19 mutant fragments. (**F**) The interactions between a series of H19 mutant fragments (H19-1, H19-2, H19-3, H19-4, H19-5) were assessed by RNA pull-down assays. Mean values ± SD, n = 3. * means *p* < 0.05, ** means *p* < 0.01.

LncRNAs usually function by physically binding to cellular factors, such as proteins [27,28,29,30,31]. Our studies showed that H19 was present in the nucleus and the cytoplasm (Figure 1A). RNA pull-down assay was performed to identify the interacting proteins of H19. The PSCs at the period of myotube formation were collected and lysed. The biotinylated H19 and biotinylated antisense H19 were incubated with PSC cell lysates, and their interacting proteins were captured by streptavidin beads and then subjected to mass spectrometry (MS). Among the different proteins detected by MS, TDP43 attracted our attention the most. TDP43, as a RNA/DNA binding protein, plays an important role in myoblast differentiation [32]. Western blot analysis was carried out to validate the interaction between H19 and TDP43 (Figure 1B). The results indicated that TDP43 was precipitated by biotinylated H19 but not biotinylated antisense H19. RIP assay was performed to confirm this interaction. As expected, TDP43 pulled down H19 in PSC lysates (Figure 1C,D), suggesting that TDP43 interacts with H19 in vivo. We performed co-localization assays to confirm the interaction of H19 and TDP43, as shown by H19 RNA FISH and TDP43 immunofluorescence staining (Figure 1A). The direct binding of H19 to TDP43 raised the possibility that H19 directly regulates the function of TDP43.

We also explored the region of H19 that was bound to TDP43. For this purpose, a series of H19 mutant fragment vectors were created (Figure 1E). Five probes containing biotin were synthesized by in vitro transcription. Pull-down and Western blot assays demonstrated that TDP43 bound to H19-2 and H19-3 (Figure 1F), suggesting that the 500-1494 base pairs of H19 are sufficient and necessary for binding to the TDP43 protein. In sum, these results indicated that H19 directly interacts with TDP43. 

### 3.2. TDP43 Promotes PSC Differentiation

**Figure 2:**
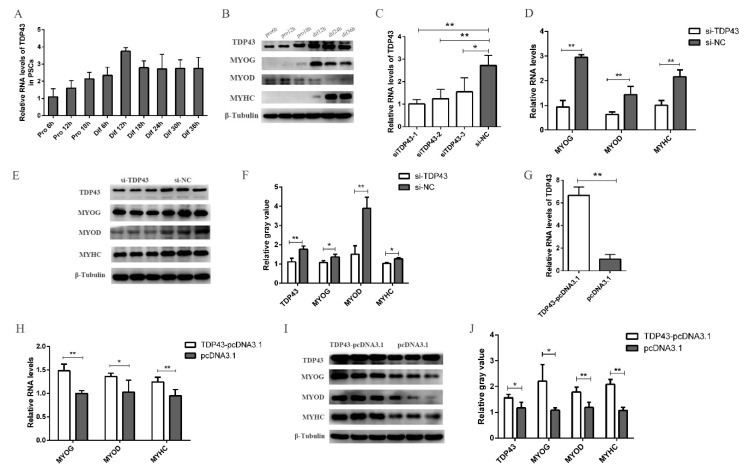
TDP43 promotes the porcine satellite cells (PSCs) differentiation. (**A**) Real-time PCR analysis of TDP43 expression in PSCs during the period of proliferation and differentiation. There was a 6h interval between each period. (**B**) Western blot analysis of TDP43, myogenin (MYOG), myogenic differentiation (MYOD), and myosin heavy chain (MYHC) protein expression levels in PSC in different periods. (**C**) The knockdown efficiency of TDP43 siRNA. (**D**) qPCR results showed that the mRNA expression of MYOG, MYOD, and MYHC were significantly decreased by TDP43 knockdown. (**E**) Western blot results showed that the protein expression of MYOG, MYOD, and MYHC were significantly decreased by TDP43 knockdown. (**F**) Quantitative analysis of Western blot results. (**G**) The overexpression efficiency of TDP43-pcDNA3.1. (**H**) qPCR results showed that TDP43-pcDNA3.1 significantly increased the mRNA expression of MYOG, MYOD, and MYHC. (**I**) Western blot results showed that TDP43-pcDNA3.1 significantly increased the protein expression of MYOG, MYOD, and MYHC. (**J**) Quantitative analysis of Western blot results. Mean values ± SD, n = 3. * means *p* < 0.05, ** means *p* < 0.01.

To explore the function of TDP43 in PSCs, we observed the expression pattern of TDP43 in PSCs at different proliferation and differentiation time points. Results showed that TDP43 had a higher expression level in the differentiation periods than in the proliferation periods, and it maintained at a relatively high level in the differentiation periods. This result indicates that TDP43 functions in the differentiation of PSCs (Figure 2A). We also detected the protein expression level of TDP43 and three differentiation markers in different proliferation and differentiation periods. Consistent with the mRNA level, TDP43 had a higher protein expression level in the differentiation periods than in the proliferation periods. Consistent with previous reports, MYOD was expressed in the proliferation periods and early differentiation periods, whereas MYOG and MYHC were only expressed during the differentiation periods. In particular, the protein level of MYHC increased sharply at the late stage of differentiation when myotubes began to form (Figure 2B).

**Figure 3:**
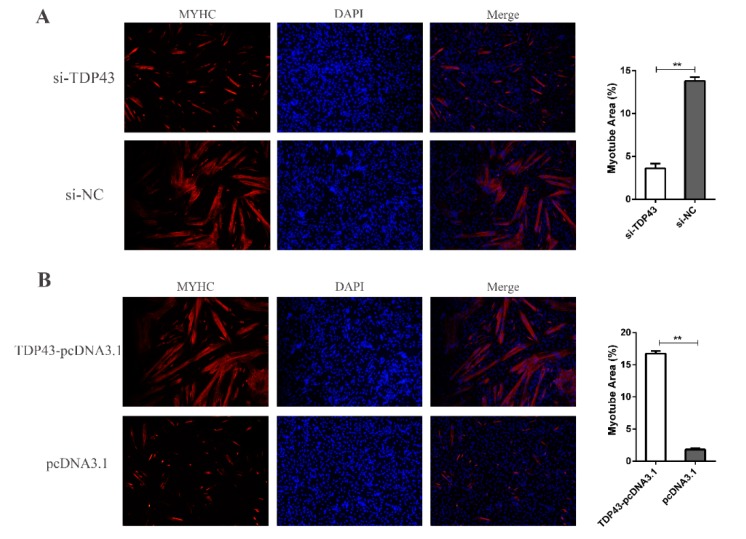
(**A**) Representative photographs of MYHC immunofluorescence staining in PSCs differentiated for 24 h showing that knockdown of TDP43 significantly decreased the MYHC expression level. (**B**) Representative photographs of MYHC immunofluorescence staining in PSCs differentiated for 24 h showing that TDP43-pcDNA3.1 significantly increased the MYHC expression level. Mean values ± SD, n = 3. * means *p* < 0.05, ** means *p* < 0.01.

In the following experiment, the three siRNAs were designed and transfected into PSCs, and the PSCs were harvested at 24 h after induced differentiation. siTDP43-1 achieved the highest knockdown efficiency of TDP43, and it was used for subsequent experiments (Figure 2C). The down-regulated TDP43 level significantly reduced the mRNA and protein levels of MYOG, MYOD, and MYHC (Figure 2D–F). To further confirm the results, we constructed an overexpression vector of TDP43 (TDP43-pcDNA3.1) to transfect PSCs. As expected, TDP43-pcDNA3.1 significantly promoted the myogenic differentiation by increasing the mRNA and protein levels of MYOG, MYOD, and MYHC (Figure 2G–J). Simultaneously, TDP43 knockdown decreased the formation of myotubes and TDP43 overexpression significantly increased the number of myotubes, as demonstrated by the immunofluorescence of MYHC (Figure 3). Collectively, our experimental data revealed the possible functional role of TDP43 in promoting myogenic differentiation.

### 3.3. H19 Regulates PSC Differentiation by Affecting the Enrichment of TDP43 at the MYOD Promoter

**Figure 4:**
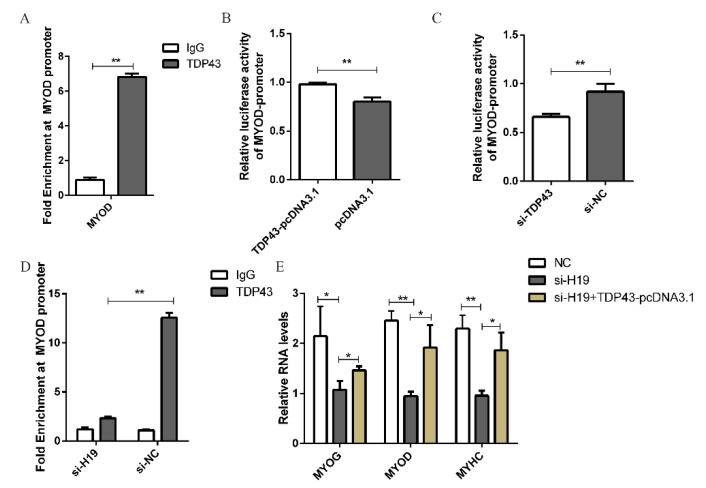
H19 regulates MYOD by affecting the enrichment of TDP43 at the MYOD promoter. (**A**) Chromatin Immunoprecipitation (CHIP)qPCR results revealed that TDP43 bound to the MYOD promoter. (**B**) and (**C**) Luciferase reporter assay of MYOD-promoter in PSCs transfected with either TDP43-pcDNA or TDP43 siRNA. (**D**) CHIP-qPCR results revealed that H19 knockdown significantly decreased the enrichments of TDP43 at the MYOD’s promoter. (**E**) Real-time PCR analysis of expression of MYOG, MYOD, and MYHC in PSCs after transfection with H19 siRNA or transfection with H19 siRNA and TDP43-pcDNA3.1. Mean values ± SD, n = 3. * means *p* < 0.05, ** means *p* < 0.01.

Previous studies have shown that TDP43 promotes MYOD expression by directly binding to its promoter in C2C12 cells [32]. In the present study, TDP43 was also enriched in the promoter of MYOD in PSCs through the CHIP experiment, indicating the same mechanism in mice and pigs (Figure 4A). Moreover, we inserted the promoter region of MYOD into the pGL3 plasmid to detect the luciferase activity. Results showed that the transfection of TDP43-pcDNA3.1 significantly increased the luciferase activity of the MYOD promoter (Figure 4B). By contrast, the transfection of TDP43 siRNA significantly down-regulated the luciferase activity of the MYOD promoter (Figure 4C). These results further indicated that TDP43 can bind to the promoter of MYOD and affect the promoter activity of MYOD.

**Figure 5:**
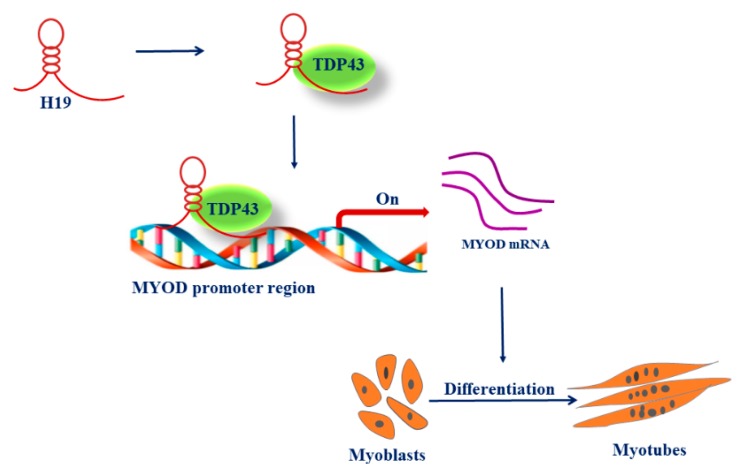
Model for H19-regulated PSC differentiation.

Considering that H19 affected the expression of MYOD in PSC differentiation, we speculated whether H19 regulates MYOD expression through TDP43. As expected, CHIP-PCR assays revealed that H19 knockdown significantly reduced the enrichment of TDP43 at the promoter of MYOD (Figure 4D). H19 siRNA and TDP43 overexpression vectors were co-transfected into PSCs to confirm whether H19 affects MYOD expression through TDP43. As expected, TDP43 overexpression partially rescued the decrease in PSC differentiation caused by H19 knockdown, as proven by the rescued mRNA expression levels of MYOG, MYOD, and MYHC (Figure 4E). The results indicate that H19 regulates PSC differentiation in a TDP43-dependent manner. Taken together, these results suggest that H19 acts as a scaffold to recruit TDP43 to the promoters of MYOD in PSCs, thus activating the transcription of MYOD, leading to PSC differentiation (Figure 5).

## 4. Discussion

H19 is one of the best known lncRNA; it is located in porcine chromosome 2, mouse chromosome 7, and human chromosome 11. H19 contains 5 exons and 4 introns [33,34,35]. Highly expressed in skeletal muscle, H19 is also highly abundant in almost all embryonic and neonatal tissues. However, H19 is dramatically down-regulated in all tissues except skeletal muscles shortly after birth. Due to the high expression of H19 in skeletal muscles, H19 plays an important role in skeletal muscle development. For example, H19 performs a critical trans-regulatory function in skeletal muscle differentiation and regeneration by encoded miRNAs in mice [25]. Xiaochun Xu et al. also demonstrated that H19 can promote the differentiation of bovine skeletal muscle SCs by suppressing Sirt1/FoxO1 in bovine [36]. Despite this published research progress, our knowledge of the genetic factors underlying the variation in PSCs and differentiation involving H19 remains incomplete. In particular, the molecular mechanism by which H19 regulates PSC differentiation needs further research.

Similar to other regulatory RNAs, lncRNAs can interact with protein factors and nucleic acids. Thus, they have the potential to direct RNA complexes to specific RNA or DNA targets [37,38,39]. lncRNAs also perform different interaction mechanisms due to their subcellular localization. For example, as a nuclear-retained lncRNA, Myoparr augments the interaction between Ddx17 and P300/CBP associated factorproteins, and promotes the Ddx17/PCAF complex enrichment into the promoter of MyoG. The enhanced Ddx17-PCAF interaction by Myoparr may be sufficient for maximum polymerase II recruitment to the MyoG promoter and further promote MyoG transcription and myoblast differentiation [40]. Different from nuclear-retained lncRNAs, cytoplasm-located lncRNAs usually have different functions. For example, lnc-31 was required for myoblast proliferation and promoted ROCK1 protein synthesis by stabilizing its translational activator, YB-1 [41]. In the present study, H19 was expressed in the cytoplasm and the nucleus. We explored the interaction proteins of H19 through RNA pull-down and RIP assays, and we focused our attention on TDP43 because of its ability to promote MYOD expression. Moreover, a series of truncated mutant fragments were synthesized and subjected to pull-down assays. The results showed that 500-1494 base pairs of H19 are sufficient and necessary for binding to TDP43. All of the above experiments demonstrate a direct binding of H19 to TDP43 protein.

TDP43, as a RNA/DNA-binding protein, plays an important role in many biological processes, such as pre-mRNA splicing, mRNA transport and stability, and transcription [42,43,44]. Meanwhile, TDP43 is important for the differentiation of myoblasts into myotubes in mice [32]. TDP43, as a member of the hnRNP family, binds mRNA and DNA to regulate mRNA splicing, stability, and translation as well as gene transcription [42,43]. Recent studies have shown that TDP43 is associated with muscle development. For example, loss of TDP43 causes muscle degeneration and is lethal in zebrafish [45]. Simultaneously, knockdown of TDP-43 reduces the expressions levels of MyoD and myogenin while overexpression of TDP-43 causes an opposite result in mice, indicating the importance of TDP43 for the differentiation of myoblasts into myotubes [32]. PSCs were the cells which were most similar to the porcine muscle state in vivo. In order to detect the effect of TDP43 on muscle differentiation, we chose PSCs as the model. Previous studies have reported that TDP43 regulated muscle differentiation in mice. We also wanted to examine the function of TDP43 in different species. In the present study, we detected TDP43 expression levels at different proliferation and differentiation time points. Results showed that TDP43 was significantly upregulated in the differentiated PSCs, indicating that TDP43 is involved in PSC differentiation. TDP43 was found to significantly promote the differentiation of PSCs by performing gain or loss functional assays. It has been reported that TDP43 can enrich in the promoter of MYOD. We also found the same mechanism in PSCs through CHIP and luciferase reporter assays, and found that H19 can affect the enrichment of TDP43 on MYOD’s promoter. The knockdown of H19 significantly decreased the enrichment of TDP43 in the promoter of MYOD and decreased the expression of MYOD. Our data support the notion that H19 acts as a scaffold guiding TDP43 to the functional sites and regulates the transcription of MYOD, further mediating PSC differentiation. The rescue experiment further verified the results.

In reviewing the results of this study, some potential limitations should be considered. Although our results showed the molecular mechanism by which H19 and TDP43 regulate PSC differentiation, more complex mechanisms in vivo may need further research. Meanwhile, the analysis of H19 overexpression could not be carried out because of the high expression of H19. Therefore, we only detected the enrichment level of TDP43 in the MYOD promoter after H19 knockdown.

## 5. Conclusions

We explored the effects of H19 and TDP43 in PSC differentiation, and our results provide a molecular explanation for the H19/TDP43-mediated enhancement of MYOD transcriptional activity in PSC differentiation.

## Supplementary Materials

The following are available online at https://www.mdpi.com/2073-4425/11/3/259/s1, Figure S1: H19 knockdown inhibited the differentiation of PSCs, Table S1: Primers used for plasmid construction, Table S2: Primers used for qPCR, Table S3: Primers used for RIP or CHIP.

## Abbreviations

PSCs:	porcine satellite cell
SCs:	satellite cells
lncRNA:	long non-coding RNAs
RIP:	RNA immunoprecipitation
CHIP:	chromatin immunoprecipitation
FISH:	RNA fluorescence in situ hybridization
TDP43:	TAR DNA binding protein 43
MYOG:	myogenin
MYOD:	myogenic differentiation
MYHC:	myosin heavy chain
MS:	mass spectrometry
siRNA:	small interfering RNA
