# Supplementary material for: Long Non-Coding RNA H19 Promotes Porcine Satellite Cell Differentiation by Interacting with TDP43
Source: Genes (Basel). 2020 Feb 28;11(3):259. doi: 10.3390/genes11030259 (PMC7140797; doi:10.3390/genes11030259)
Supplement: Supplementary file 1 [file genes-11-00259-s001.pdf]

Supplementary Figure

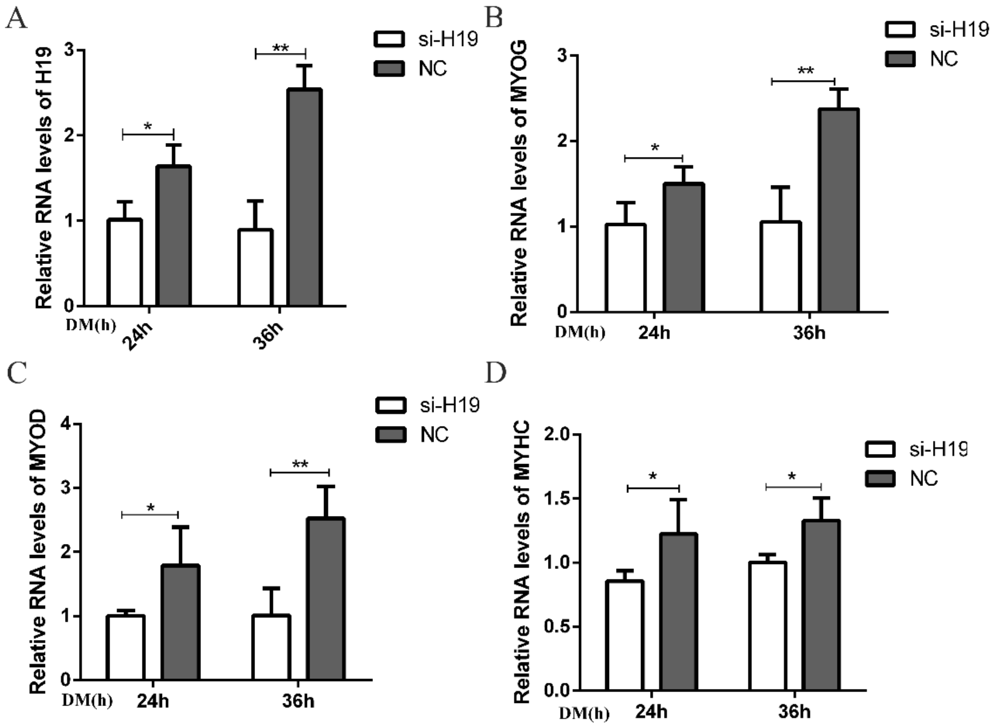

Figure S1. H19 knockdown inhibited the differentiation of PSCs.(A) The knockdown efficiency of H19 siRNA. (B),(C) and (D) qPCR results showed that the mRNA expression of MYOG, MYOD, and MYHC were significantly decreased by H19 knockdown.

Supplementary Table 1. Primers used for plasmid construction

Primer name	Primer sequence(5′-3′)
TDP43 full-length primer	F: TTCCCTGTCGGGCTTCCTA<br>R: CCAACCATAACCCAACGTC
MYOD promoter primer	F: ATGCCAACTCCTCTCCTAAAC<br>R: GGAAGCAGCCAATACAAAAG
H19 full-length sequence	F: AAGCAGGGTGAGGGAGGGGGGTG<br>R: TGAGTTTATTGATGAGTCCAGGGC
H19 1-600bp fragment	F: AAGCAGGGTGAGGGAGGGGGGTG<br>R: GCCGCCACTCTGCCGGGGGGTCAG
H19 500-940bp fragment	F: TTCCCTCCCCAGGCCTTGTT<br>R: ACTGCACCTTCTTTCCCATTCTCC
H19 915-1494bp fragment	F: CGGAGAATGGGAAAGAAGGTG<br>R: AGCCGAGAGTGTTCAGGAAGGC
H19 1442-1887bp fragment	F: TGACGGAGAGGGACAGACGTGA<br>R: GACAGAGGAAAAAAAGGAGGAAGGG
H19 1863-2272bp fragment	F: CCCTTCCTCCTTTTTTTCCTCTG<br>R: TGAGTTTATTGATGAGTCCAGGGC

Supplementary Table 2. Primers used for qPCR

Gene	Primer sequence(5′-3′)
TDP43	F: AAATGGATGAAACAGATGCC<br>R: TCCGTAAAACGAACAAAGC
MYOG	F: ATGAGACATCCCCCTACTTCTACCA<br>R: GTCCCCAGCCCCTTATCTTCC
MYOD	F: GGCTGCCCAAGGTGGAAATC<br>R: TGCGTCTGAGTCACCGCTGTAG
MYHC	F: GTTCAGAGAAAGGCATCCCAAA<br>R: GAGAGTGACCGACACCACAAGTG
18S	F: TCCCGACGTGACTGCTC<br>R: GGTGACAGCGGGGTGG

Supplementary Table 3. Primers used for RIP or CHIP

Gene or Primer name	Primer sequence(5′-3′)
H19-RIP primer	F: GAGTGGTCCTGATGTGGTAGTGG<br>R: GCCGTCGGCTGTAATTTGAT
SnRNA primer	F: GGGAGATACCATGATCACGAAGGT<br>R: CCACAAATTATGCAGTCGAGTTTCCC
MYOD-CHIP primer	F: CAGAGGAGGGGCTGTTGAA<br>R: GGGAGATGTAGGAGGCAGTAAA
